# A Robotic Test Rig for Performance Assessment of Prosthetic Joints

**DOI:** 10.3389/frobt.2021.613579

**Published:** 2022-03-07

**Authors:** Appolinaire C. Etoundi, Alexander Dobner, Subham Agrawal, Chathura L. Semasinghe, Ioannis Georgilas, Aghil Jafari

**Affiliations:** ^1^ Bristol Robotics Laboratory, Department of Engineering Design and Mathematics, University of the West of England, Bristol, United Kingdom; ^2^ Department of Mechanical Engineering, University of Bath, Bath, United Kingdom

**Keywords:** prosthetics, prosthesis, robotic device, knee joint, velocity control, robotic joint, test rig design

## Abstract

Movement within the human body is made possible by joints connecting two or more elements of the musculoskeletal system. Losing one or more of these connections can seriously limit mobility, which in turn can lead to depression and other mental issues. This is particularly pertinent due to a dramatic increase in the number of lower limb amputations resulting from trauma and diseases such as diabetes. The ideal prostheses should re-establish the functions and movement of the missing body part of the patient. As a result, the prosthetic solution has to be tested stringently to ensure effective and reliable usage. This paper elaborates on the development, features, and suitability of a testing rig that can evaluate the performance of prosthetic and robotic joints via cyclic dynamic loading on their complex movements. To establish the rig’s validity, the knee joint was chosen as it provides both compound support and movement, making it one of the major joints within the human body, and an excellent subject to ensure the quality of the prosthesis. Within the rig system, a motorised lead-screw simulates the actuation provided by the hamstring-quadricep antagonist muscle pair and the flexion experienced by the joint. Loads and position are monitored by a load cell and proximity sensors respectively, ensuring the dynamics conform with the geometric model and gait analysis.

**Background:** Robotics, Prosthetics, Mechatronics, Assisted Living.

**Methods:** Gait Analysis, Computer Aided Design, Geometry Models.

**Conclusion:** Modular Device, Streamlining Rehabilitation.

## 1 Introduction

During the last decade or more, prosthetic functionality has advanced considerably, to close the gap between previously natural and a replicated artificial gait. This is partially due to the increased commonality of amputations (Moxey et al., 2011), while a higher functionality of the replacement joint has been associated with a better rehabilitation and mental health improvement in terms of comfortability and long term usage (Ziegler-Graham et al., 2008). The significance of a comfortable, functional prosthesis to amputees cannot be overstated. An artificial limb or joint can form the basis on which a user can start rehabilitation, enabling them to live fuller, more productive lives. The impact of prosthesis on patient satisfaction has been investigated previously (Choi and Ra, 2016) and the results show that the vast majority of subjects reported improvement given the functionality regained after a total knee arthroplasty (TKA). This is relevant as, in the U.S., TKAs are expected to increase to 3.48 million per annum by 2030, an increase of 601% since 2005 (Kurtz et al., 2007). Given the number of transfemoral amputations and a prostheses’ impact to the quality of life of a patient, assessing the utility, quality and comfort of new and existing prosthetic devices is paramount.

This has spurred research into investigating different aspects of optimum design, functionality and performance of transfemoral devices, as there are numerous mechanical principles that could be employed to moderate the inertia experienced by the amputee (following increase in stability/reduction of jerky movement at the knee). Some prosthetic devices use fluid-based resistance from hydraulic and pneumatic systems controlled by electronic components whereas some others make use of electrical motors. In addition to the ability to control the articulation, the geometry also needs to adhere to the parameters of a natural gait, which can be achieved through biomimicry. This work led to the development of prosthetic joints that better emulate the normal biomechanics of a human gait through artificial condyles and tendons, obtaining movement closest to the regular compound motion usually exhibited by the knee (Etoundi et al., 2013).

The design and development of the prosthetic joint itself is only part of the manufacturing process for a fully conceived solution as the joint itself has to be tested stringently in parallel and before its use. Testing of such devices need to undergo two regimes. On the one hand, this knee must be tested in clinical trials, with the complexities of subject attraction and trialling untested devices which could cause discomfort, pain or injury. In addition to the human testing, mechanical testing must be performed in order to determine whether these devices can withstand the typical life cycle of an amputee. A functioning test apparatus appropriately equipped with sensors that could also measure parameters of interest which can be difficult to measure directly in a test subject. Simulations and modelling approaches can only take us so far down the design process; therefore, a physical testing system is a necessity.

Robotic testing has emerged as a practical approach to conduct repeatable dynamic testing to evaluate and compare prototypes especially with sensory feedback that can not be obtained through human testing. The minimum two main variables of any gait emulator are the hip to foot vertical displacement and the resultant angle between the tibia and femur, as much of the motion occurs in the sagittal plane (Richter and Simon, 2014). The setup of the robotic testing platform would have to be bespoke to the joint tested, meaning the facility would have to be modular in design. To emulate the articulation within the joint, the lead-screw controlled by motor causes flexion at the joint and which if repeated imitates a walking gait. The equipment needed to create and monitor an asymmetrical gait flexion profile, meaning the ability of recreating both the swing and stance stage was vital to the facility’s validity. In order to verify whether or not the dimensional criteria has been fulfilled, sensory components were integrated to monitor various parameters around the rig i.e., load cells monitor the axial force going through the prosthesis, proximity sensors control and monitor the position of the lead-screw with cameras providing a visual capture of the rig moving whilst under the interlocked protection of the safety cage. These ensure that there is no damage to components during testing.

Several examples of a device of this type, elaborated on in Section 2, have been used to demonstrate the viability of this method, and this paper discusses the recent example designed for multiple types of prostheses using the angle of flexion and desired cadence from previously analysed gait. When implemented with a passive prosthesis or single-axis joint often utilised in robotics, the geometry can be modelled as a multi-linked system, actuated *via* rotation from one position, inducing linear movement along the vertical displacement, with the prosthetic providing a damping component. To emulate any kinematic gait, controlling the input to the actuation is necessary, as the remaining variables such as load on the various members including ground reaction force is covered by sensors or calculated and extrapolated from the mathematical model.

The paper is organised as follows: Section 2 denotes the analysis conducted to determine to the optimum construction and the methods involved in the creation, development and control of this novel platform; Section 3 displays the time-domain control results of limited testing; Section 4 discuss the implications, pandemic hindrances and recommendations to develop the system further.

## 2 Materials and Methods

From the literature (Maletsky and Hillberry, 2005; Yildirim et al., 2009; Ding et al., 2011; Wünschel et al., 2013), the characterisation of human joints locomotion is fundamental for the testing of prosthetic joint. Over the last 30 years, several testing facilities have been designed and developed to duplicate the human behaviour in order to assess the performances of rehabilitation devices under particular loading and conditions.

### 2.1 Gait Analysis

The behaviour of an active lower limbs amputee (hence known as Subject A) was investigated to define the operational parameters of the testing platform. The test rig was designed to test various motions (walking, running, jogging, etc.) of the knee joint. We know for a fact that an amputee triathlete has a clear distinction of the type of locomotion required when they are competing. They go through various specific motions in exaggeration compared to a normal amputee, hence, pushing the limits of the joints and also replacing the joints based on the need of the specific activity. Therefore, a triathlete amputee is a perfect candidate for testing the joint.

**FIGURE 1:**
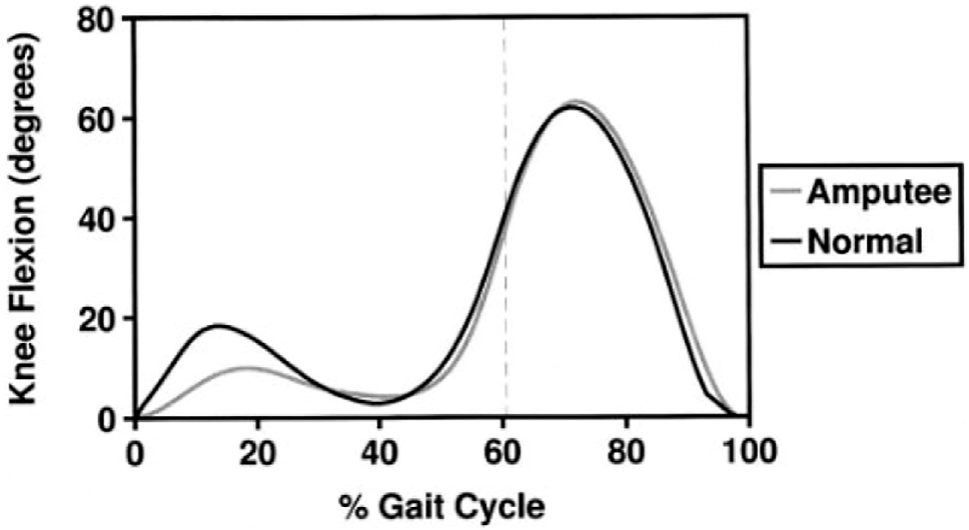
Comparison of knee flexion between a normal and amputee gait (Powers et al., 1998).

As autonomous as it can feel, walking requires the delicate control of multiple entities each exerting various angular and linear velocities, repeating cyclically along the smooth path of centre of gravity continuing iteratively (Radcliffe, 1962). There is a wide range of motion that the knee can perform, with many permutations of flexion, abduction and load whilst performing various activities (D'Lima et al., 2012). The unique parameters that make up this motion is known as a person’s gait, tracked over the full cycle between when one foot leaves the ground, it comes back into contact and once again begins to leave the ground, and recording all the periphery kinematics can aid in the analysis of said gait. These kinematics will vary from person to person, but this is especially pertinent if applied to someone who has experienced limb loss. As seen in Figure 1, there are two maximums or peaks in flexion; one at the Early Swing Stage (ESS) at around 70% of the gait cycle where the leg straightens during forward motion and one in the Early Single Support Stage at around 15% of the gait cycle where the leg is in contact with the ground, possibly due to common *trans*-femoral amputee deviations such as lateral trunk flexion as they tend towards their artificial limb. Initially, in order for the joint to perform locomotion, due to the geometry and arrangement of the test rig, the joint needs to be unlocked however this creates some unwanted deflection in the peak values. The emulation of this gait is important, but a case could be made that the Early Single Support Stage flexion peak is less significant for the cyclic testing due to its magnitude and the aforementioned reason, hence, it was disregarded as it would not affect the reliability of the products tested.

**FIGURE 2:**
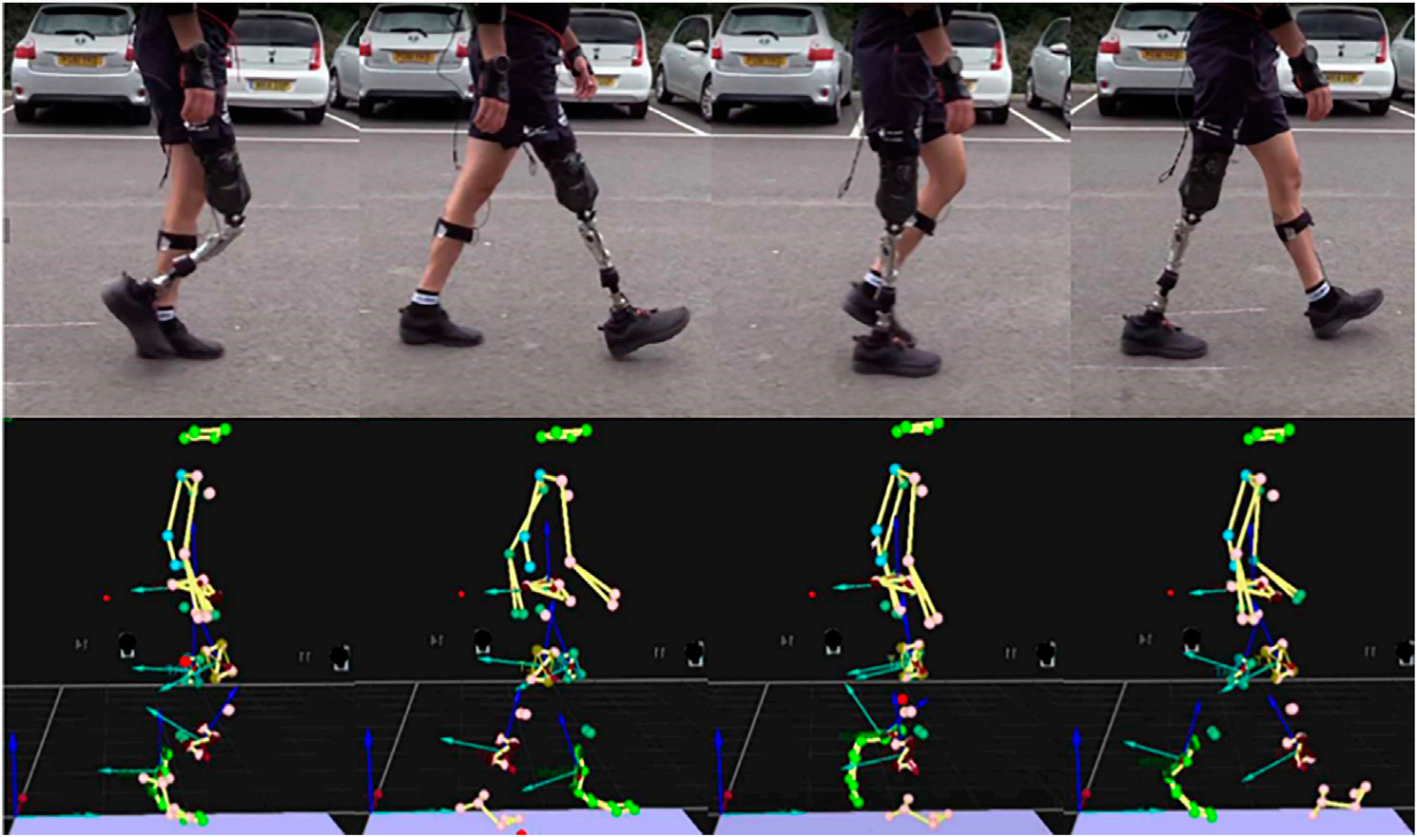
**(top)** Subject A participating in kinematic study by walking **(bottom)** Motion capture results showing typical gait (Dobner).

A kinematic study of Subject A performing various movements was conducted using an Xsens motion capture system. Sensors were placed on Subject A’s major body segments for recording the motion along with the video recording at different angles for post-analysis. Subject A was a perfect candidate as they regularly participate in triathlons, which includes a variety of motions that the platform could emulate as multiple sports are involved in the competition (Etoundi et al., 2013). Using motion capture, several variations of motion were recorded and quantified, but initially the focus was on walking because if this could be successfully imitated in the rig, the project could progress to faster forms of movement such as jogging, cycling and running. Two studies were conducted into the kinematics of Subject A’s walking, one with a carbon fibre blade connected to an Ottobock 3S80 performance joint used for his more athletic activities and his everyday prosthetic leg involving the Ottobock 3R106. As a reputable manufacturer, these prostheses formed a good starting point as they were two different types of joint – single access and polycentric, respectively. Further visual tests with the Ottobock knee were carried out to highlight the gait, shown in Figure 2.

**FIGURE 3:**
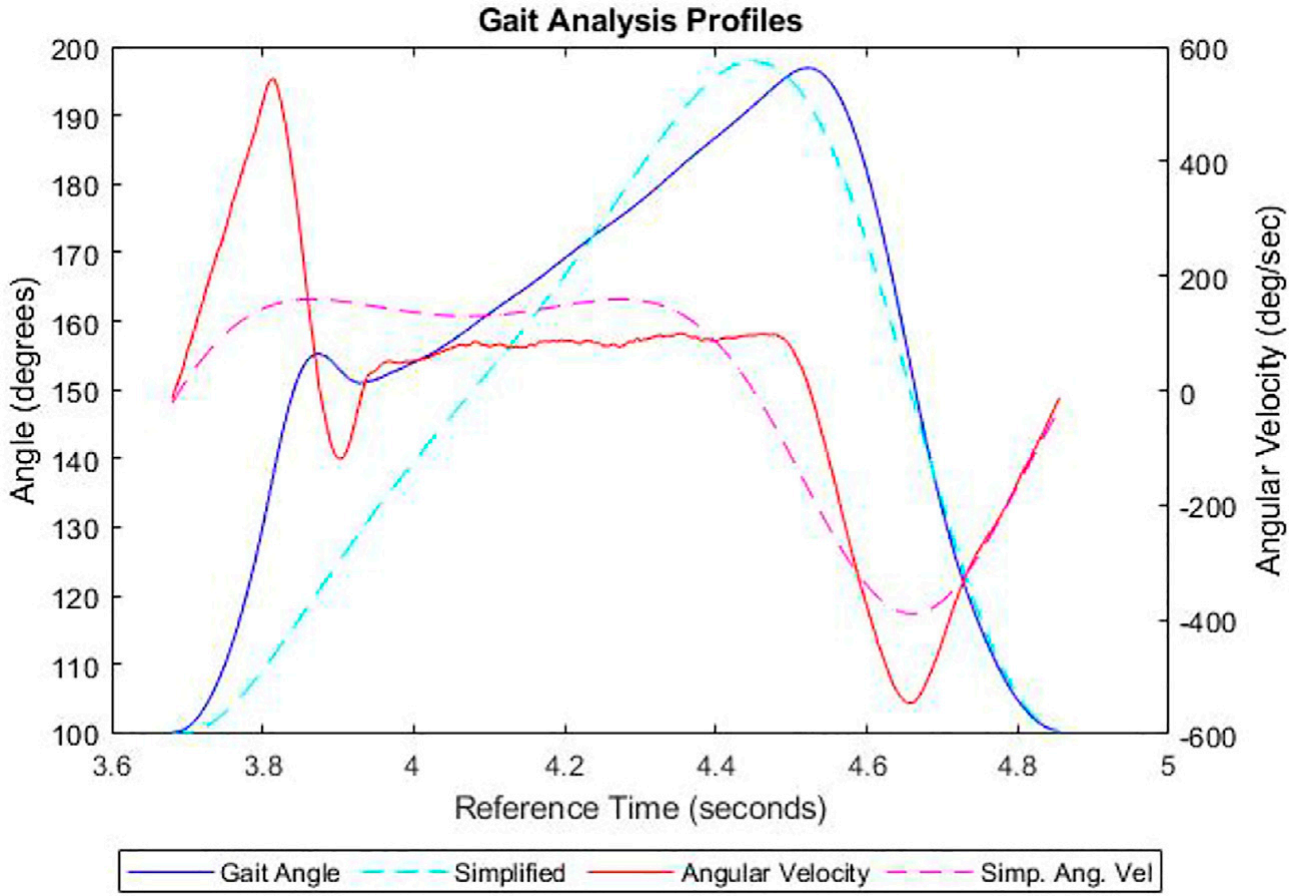
The flexion angle and associated angular velocities of the human knee joint along with their simplified plots.

Five sensors were used in this study, placed on the pelvis, the left and right thighs, the left shin and the prosthetic itself. These sensors each recorded the local position of the body part in three dimensions and the rotation in three dimensions, capturing 200 data points per second which meant incredible detail could be caught, subject to the accuracy of the sensors. Since the locomotion for above-knee amputees is subject to the level of amputation, Subject A had an ideal level of amputation that enabled him to undertake all the activities required in this study. Also, the level of amputation is taken care of by the personalized socket and the position where the prosthetic knee joint attaches is basically the same for all above knee amputees which allows us to generalize our study for the larger above knee amputee population. A graph could then be produced of the flexion data as shown by Figure 3, which revealed his average cadence to be 1.18 s per stride or approximately 50 strides per minute which was considered plausible target. Although the data observed possible hyperextension of the leg, the maximum and minimum flexion angle could also be obtained and a sensible range of approximately 90–170, respectively, which falls within the manufacturer’s quoted specifications. These will play a part in the amplitude, period and vertical shift of the wave signal for the motor to mimic this analysis, including number of cycles. With the gait analysis, there are differing angular accelerations of the knee either side of the double-support phase represented by the varying gradients in the asymmetrical oscillation, which was rectified using a modified nested sine function to ensure the atypical sinusoidal gradients were adhered to sufficiently. It can also be represented as the function shown in Equation 1, where theta is the angle between the femur and tibia, lambda is the gait period with A1 and A2 being the minimum and maximum amplitudes, respectively. Since an emphasis was placed on the achieving the articulation, as the ground reaction force can be manipulated with added weight, the midstance peak in flexion angle has been ignored as it fell in the maximum range.

$$\theta_{\mathrm{fl}exion}\left(t\right)=\frac{A_{1}+A_{2}}{2}+\frac{A_{1}-A_{2}}{2}\sin\left(\frac{2.\pi }{\lambda }t-\frac{\sin\left(\frac{2.\pi }{\lambda }\right)}{2}\right)$$ (1)

### 2.2 Device Manufacture

**FIGURE 4:**
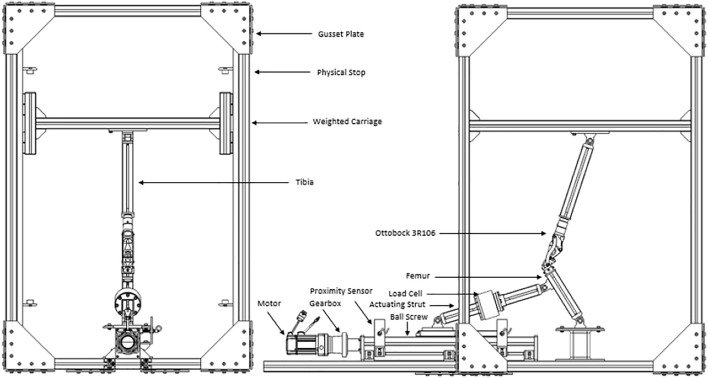
Schematic of the mechanical components (Dobner).

The robotic platform was constructed with the principles of modularity and absolute safety, with a schematic of the device shown in Figure 4. Based on an aluminium profiled framework, a geometry envelope is created to contain the dynamic assembly, with an interlocked safety cage on the exterior to protect any users. Gusset plates also provide reinforcement to prevent movement whilst in operation and are found in all corners on the six faces of the hexahedral frame. These bolted gusset plates sit in a recessed floor with loadable ballast boards, to prevent further movement during operation in both lateral and vertical directions, respectively. Vertical movement is created by translating rotational motions to linear motion to emulate the hip displacement from the ground. A brushless AC servo motor (Applied Motion J0750-302-5) is directly connected to a linear ball screw actuator (HepcoMotion SDM30100) with a pitch of 20 mm/rev, *via* a gearbox (Wittenstein NP015S-MF1-10-1) to aid torque augmentation. The motor communicates with the servo and microcontroller to enable transfer of the correct signals required to drive the motor dependant on the platforms state. The total travel of the ball screw is 270 mm with reasonable safety limits, and to ensure no damage occurs, the travel is appropriately bookended by a pair of proximity sensors acting as limit switches. An absolute position reference of the ball screw runner is established by an encoder mounted inside the electrical panel, which then determines the other positions of the joints based on the device configuration setup. Pin joints with 180-degree articulation allow the other members to move freely in accordance to the setup, with threaded fittings held by suitable adhesives to ensure modularity. A planar platform guided by vertical rails is centrally attached to the upward-most strut to safeguard unequal wear on the rails and bearings. Physical stops have also been implemented as the motor is non-locking, to prevent the carriage falling and damaging the hardware during a power failure. There are several other safeguards in place including but not limited to the lock box and breakers to the electrical supply and servo. Prostheses are attached using bespoke adaptors to connect the profiled struts to either extremity of the joint *via* interference fit. The knee joint is inverted to be able to adjust the ground reaction force accordingly to the patient weight described, dependant on the finite element analysis of the joint and accompanying calculations. Safety concerns negated the viability of an upright configuration as the velocities required for the various components of higher weight would present a severe hazard to the user. As a result, the actuation from the static ball screw acts as the quadricep-hamstring antagonist pair, enhancing the biomimicry of system. A load cell is implemented on the actuating arm to monitor the load going into system. All instrumentation is managed by the National Instruments myRIO microcontroller, operated with a PC and the relevant software.

#### 2.2.1 Computer and Geometry Models

**FIGURE 5:**
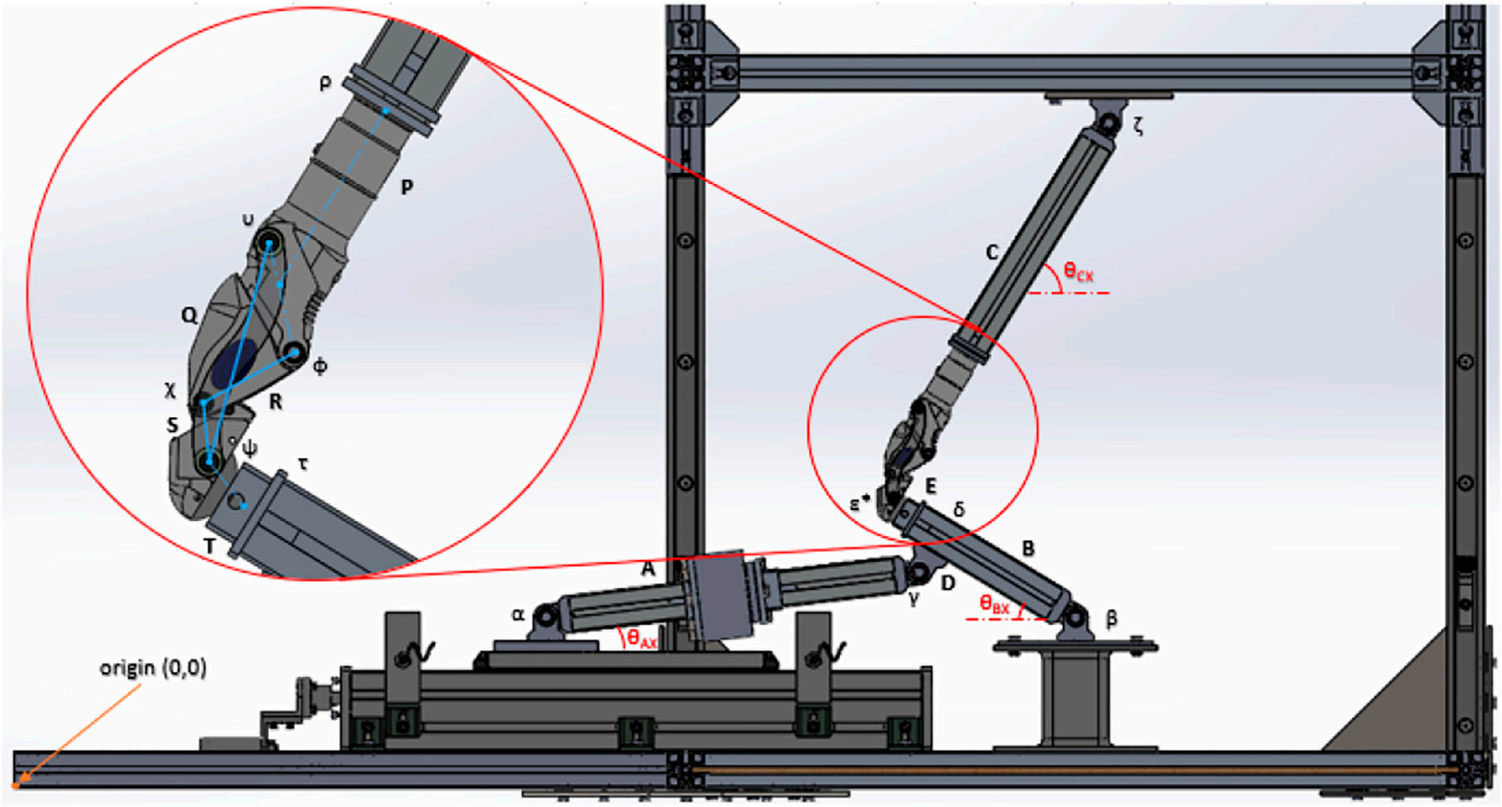
Diagram of the Cartesian method of the Geometry Model for the Ottobock 3R106 (Dobner).

CAD models initially developed to visualise the design of the rig preventing any obvious interference or obstruction and was updated proficiently for use by the industrial collaborators (Dorset Orthopaedic Ltd, Ottobock Ltd.) on the project. The design project was then simplified into a geometry model to check viability of various permutations and allow simulations in a virtual environment which was then verified using a parametric study with no joints involved. By using the virtual model, the necessary trajectories where tested and implementation of different knee joints tested. As an example, an Ottobock 3R106 was modelled from an original using an Aberlink Coordinate-Measuring Machine (CMM) and then mapped as an interlinking two-dimensional system, appropriate as the knee is a uniaxial diarthrosis. Once inserted into the system to check viability, a spreadsheet-based geometrical model of both the prosthesis and rig was created to quantify the validation method used here. Using Grashof evaluation criterion (Etoundi and Burgess, 2014), the polycentric prosthesis in question is classed as a double rocker of a high aspect ratio, shown by Figure 4, with one of the axes mounted higher than the other to provoke more rotation. A four-bar linkage of this kind emulate the biomechanics in a biological knee as the ligaments control the translation and rotation between the adjacent elements. As a result, the scalene trapezoid formed by the four-bar linkage transforms from acute to crossed when going from minimum to maximum flexion, which creates multiple states of trigonometry to calculate the various positions, but these have been accounted for. Designating each node and link with appropriate notation, an example shown in Figure 5 allows correct triangulation of each position and angle dependant on the geometric state. This feature enables a set of desired parameters to be entered in such as the limb lengths and the desired flexion and it will evaluate motion and output a Boolean response indicative of plausibility.

### 2.3 Control

The control of the platform is performed by two pieces of software; SVX Servo Suite supplied with the motor for basic control of the motor and servo, and NI LabVIEW to control the inputs and outputs from the myRIO. Within LabVIEW, the Express Virtual Instruments were configured to communicate correctly with the pin connections on the myRIO and in turn the motor’s parameters such as the motor behaviour, sensory response, or control of the program itself depending on user requirements. The SVX Servo Suite sets the mode of the motor manipulated by the LabVIEW program, and for initial testing Analogue Velocity Mode (AVM) and a value of 10 rev/sec/V were chosen for its simplicity in troubleshooting. A simplified gait wave, as mentioned in Section 2.1, was modified into a function of angular velocity of the motor using the spreadsheet tool which then can be inputted using numeric controls to create a gait similar to subject A’s. By fine-tuning control scheme that confirms the correct velocity or scalar motor parameter, the more accurate the emulation within the platform. Within the LabVIEW program, safeguards were also implemented to safeguard against any improper use. As a result, a SR-type flip flop was installed in conjunction with the proximity sensors to ensure the motor would not overreach the limits of travel of the ball screw. This ensures that the direction of the motor will change once the metallic runner interacts with the inductive proximity sensor. Feedback of the motor direction and velocity is then returned to the PC. A fault condition is also imposed to stop the program preventing incorrect signals from doing any damage, such as both sensors triggering simultaneously. A counter was also introduced in order to fulfil the overall device’s need to perform testing over an extended period of time. This was achieved through circumstantial programming by observing the change in the proximity sensor states and counting them under certain conditions, ignoring incorrect events such as consecutive single sensor triggers.

### 2.4 Regulatory Compliance

An important consideration in the rig’s utility is the ability to perform the tests according to the criteria of regulatory bodies. As the rig is meant for the testing of lower limb prosthetics, it needs to be able to prove that these devices are suitable by subjecting them to a variety of conditions, both static and dynamic (British Standards Institution, 2016). The implementation of such test systems is governed by ISO standard Prosthetics—Structural testing of lower-limb prostheses—Requirements and test methods (ISO 10 328:2016) which sets out the following key criteria:

• Principal static and cyclic tests for all components
  • static test procedure consists of proof test and ultimate strength test. this test is carried out to determine the performance of the load-bearing structures under typical severe loading conditions that can occur during use by users as occasional single events.
  • cyclic test procedure consists of repeated applications of a prescribed load to a test sample with loading conditions typical of normal walking, followed by a final static test for which the loading and unloading procedures of the relevant static proof test apply
• Separate static ultimate strength test in maximum knee flexion on knee joints and associated units
  • Users can apply high loads to prostheses in full flexion when kneeling or squatting (deep knee bend). A structural test is thus required, in order to ensure an adequate level of safety during normal use.
• Separate static and cyclic tests on knee locks for all mechanisms which lock the knee joint in extended position
  • Locked knee units are subject to flexion loading during the stance phase of walking, and a failure of the knee lock mechanism during this phase is potentially hazardous. A structural test is required in order to ensure an adequate level of safety during normal use.
• Basic tolerances related to accuracy of equipment and accuracy of procedure:
  • For the accuracy of equipment:
    • linear dimensions to an accuracy of ±0.2 mm,
    • angular dimensions to an accuracy of ±0.2°,
    • test forces and moments to an accuracy of ±1% of the highest value required in the test, and
    • the frequency of cyclic tests to an accuracy of ±1% of the test frequency used
• For the accuracy of procedure:
  • Linear dimensions, except segment lengths, shall be initially set and finally adjusted with a tolerance of ±1 mm.
  • Segment lengths shall be set with a tolerance of ±2 mm.
  • Angular dimensions, except the angular “toe-out” position of prosthetic feet, shall be set with a tolerance of ±1°.
  • The angular “toe-out” position of prosthetic feet shall be set with a tolerance of ±3°.
  • Static test forces and moments shall be applied with a tolerance of ±2% of the highest value prescribed for the test.
  • Pulsating test forces Fc(t) shall be applied at the instant of Fcmin with a tolerance of ±25 N and at the instant of Fcmax with a tolerance of ±3% of the value prescribed for Fcmax.
  • The frequency of cyclic tests shall be controlled with a tolerance of ±10% of the test frequency used.
  • The distance LBT between the load application points or the displacement *δ* of the moving load application point shall be controlled with a tolerance of ±1 mm
• List of equipment for principal tests and separate tests
  • end attachments required for specific set-ups of test samples;
  • a special jig that may be used on an optional basis to facilitate the setting, adjusting and/or measuring of segment lengths and offsets of test samples;
  • any devices used to measure loads and dimensions.

These criteria then formed the basis of the design considerations of the proposed test rig. The rig can perform the necessary cyclic testing under the accuracy and reliability requirements stated. The removable platform added to raise the pin joint at the centre of the frame floor was added to ensure viable articulation and geometrical validity.

Using existing geometry models and spreadsheet tools (Dobner, 2019), a configuration setup was validated with the initial parameters extracted from the ISO standard. The testing conditions including the lengths of the various struts for the principal tests (British Standards Institution, 2016). Only combination B is plausible as it is aligned with the configuration of the current rig, one with a strut either side of the knee, connecting top and bottom, which states a total leg configuration length of 650 mm with 150 for the femur and 500 for the tibia. It denotes that these parameters can be increased but the conditions for the principal tests would have to recalculated in accordance with the new values, but this would depend on the viability given by the geometry model.

Another compliance condition is the load that the prosthetic must support, which negated the need for the advanced gait analysis as it would have to be subjected to this regardless. To specify the certain load for a particular test, different loads have been categorised into testing load levels and conditions (British Standards Institution, 2016). Testing load levels signify the different classes of the various forces based on locomotion data from amputees with body mass of less than 60 kg. Other classes of forces which are more than 150 kg are also present but there are complications associated with weight and *trans*-femoral prosthesis which need to be considered when setting up a test load of this magnitude. Each class has two testing load conditions which are the maximum loading occurring early and late, which refer to both peak forces in ground reaction, during the single support phases of gait (British Standards Institution, 2016).

There are several different force designations all revolving around the load cycle that the knee must perform. The ISO standards for testing condition state that there is a minimum and maximum test force that it must oscillate between, similar to the calculated gait analysis, but contrarily the oscillation is symmetrical like a normal trigonometric way, with omega related to the cadence of the gait to be emulated. As the kinematic of the testing platform can be interpreted as a 2D truss problem, the force exerted on the knee can be used to calculate the velocity profile required to fulfil this forcing function criterion. Although there is only one load cell present in the platform’s current state, space for a second has been facilitated within the micro-controller and adjoining electrical schematics as having explicit load values either side of the joint will yield a better understanding into the unknown inefficiencies such as friction and the eventual wear in the joint.

It would take roughly 29 days of continuous running at Subject A’s cadence to achieve 3 million cycles of testing on the proposed test rig, equivalent to 2,138 km of real world running. Once the platform is configured comprehensively, it must perform these three million cycles in order to satisfy compliance, essentially emulating the life-cycle of the joint in a concentrated period, which can be monitored *via* the software.

In addition to the loads, there are lateral offsets at which the load must be applied (British Standards Institution, 2016), specified for each permutation of loading level and condition, as each loading condition represents the peak force levels at two different positions on the foot during the gait cycle. This is the reason for modularity within the rig as there are various parameters that will differ per weight class and prostheses, so the ability to alter the rig easily means its functionality is not limited. The fixings on the inboard struts and joint fixing plates, bottom (frame) and top (platform), can be loosened, moved and then re-tightened with bespoke adaptors in line with commissioning procedure. However, with the application of force coming from combined lateral offsets, there is a possibility of torsion within the pin joints which needs to be anticipated.

Due to the device’s modularity, the flexion angle between the femur and tibia can be represented as the function shown in Eq. 1, where lambda is the gait period with A1 and A2 being the minimum and maximum amplitudes, respectively. The principal structural tests include the static proof test, the static ultimate tensile strength test and the cyclic test, possibly the most prominent for this rig. The proof test will involve setting up the offsets, and placing a stabilising test force then a settling test force on the knee to create a datum, increase the force to the proof testing level according to the procedure found in the ISO standards, at which point you determine if there has been any movement from permanent deformation to which falls within a specified level, monitored with a rosette strain gauge. The ultimate strength test is similar with the variation being the increase in load, as well as a feedback loop on the procedure, seen within the ISO standard (British Standards Institution, 2016).

The measurement and accuracy are crucial parts of a product validation as although the product has been put through its paces, that assessment might have been derived from incorrect data. The ISO standard (British Standards Institution, 2016) lays out the basic tolerances of both the equipment to be used, such as the load cell, and the procedures to follow, such as the cyclic frequency inputted and produced. Appropriate error analysis can easily be performed and documented inline with the standard.

## 3 Experimental Results

The results of the three initial tests done on the real test rig are displayed below. One last test was conducted under the inquiry of what affect an increasing voltage has and how will the motor perform at different speeds. To prove the initial functionality, the limit switches were used to oscillate the direction, inhibiting the flexion of the joint to approximately 30°. The signal from the load cell had a level of noise that was mitigated in the data analysis phase, by using a Savitzky-Golay filter with a numeric window of 25 data points in the third-order polynomial. This smoothed the load profile sufficiently to be able to compared to other characteristics from the experiments undertaken on the 3R106 under the ISO regulations as described in the previous section.

Following the initial tests, a trial was conducted to the operational limits of the motor to assess the practical capability of the overall system.

Preliminary experiments were conducted to ensure the apparatus involved performed nominally before expansion to experiments involving gait emulation.

The results initially showed a close relationship between the load and joint angle depicted within Figure 7, which continued as the frequency of cadence increased, clearly showing an irregular but distinguished peak. Whilst the peaks heighten with the increase of speed as expected, the maximum load behaves unexpectedly as the peaks of the load are not concurrent with the flexion peaks at higher speed. This is likely due to the noise from the load cell which could be resolved with a signal filter. When examining the test of increasing cadence in Figure 8, the previous conclusion is reinforced, for when the ball screw runner is approaching the proximity sensor, the load increase significantly under the compression of the carriage weight and acute angle of actuation. Through direct comparison of the cadences, we find that the fastest cycle in Figure 9 took 3.6 s, a third of the velocity required to emulate the gait exhibited by Subject A. This will need to be rectified in order to emulate the gait, however there is no time constraints within the standards themselves.

**FIGURE 6:**
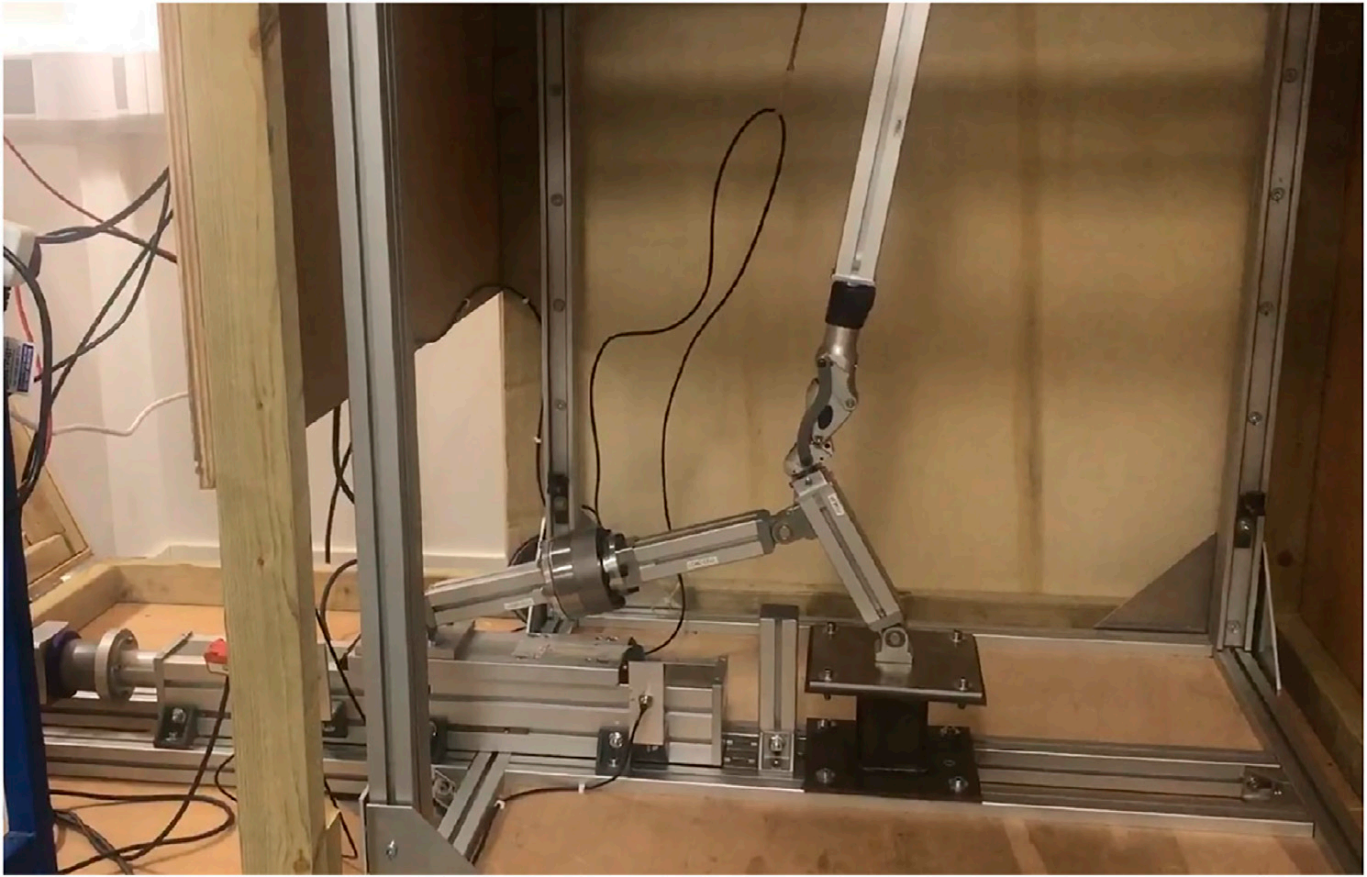
Actual test rig in motion with an Ottobock 3R106 joint (Dobner).

**FIGURE 7:**
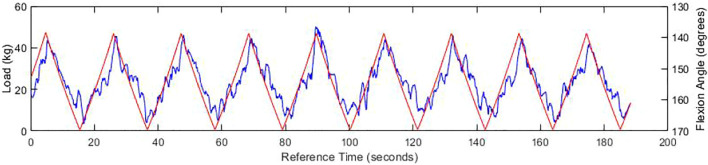
Graph of experienced load (blue) and flexion angle (red) at 1.1 V, with an SG filtration of the load function.

**FIGURE 8:**
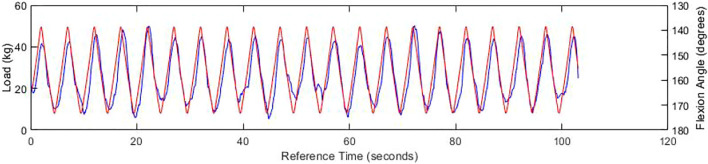
Graph of experienced load (blue) and flexion angle (red) at 6 V, with an SG filtration of the load function.

**FIGURE 9:**
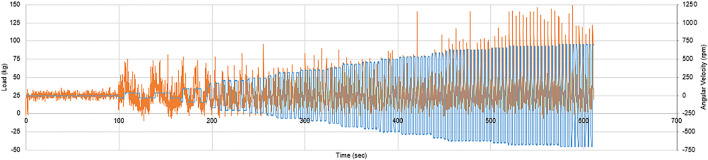
Graph of experienced load and angular velocity during a voltage increase from zero to approximately 8 V.

## 4 Conclusion

This paper was focused on the development, features, and suitability of a testing rig that can evaluate the performance of prosthetic and robotic joints *via* cyclic dynamic loading, as both enable essential complex movements. o establish the rig’s validity, the knee joint was chosen as it provides both compound support and movement, making it one of the paramount joints within the human body and an excellent subject to ensure the quality of vital replacement prosthesis. Both the loads and position are monitored by an adjacent load cell and proximity sensors respectively ensuring the dynamics conform with the geometric model and gait analysis. As part of the future work, Infrared cameras will be positioned to remotely inspect wear and proper operation in long-term testing like those necessitated by international quality standards. This work is the continuation from the design of a modular rig (Hoh et al., 2020) and has enormous potential regarding the streamlining of the testing phase of new prostheses as well as gaining insights into the design of future bio-inspired limb joints.

## Data Availability

The original contributions presented in the study are included in the article/Supplementary Material, further inquiries can be directed to the corresponding author.
